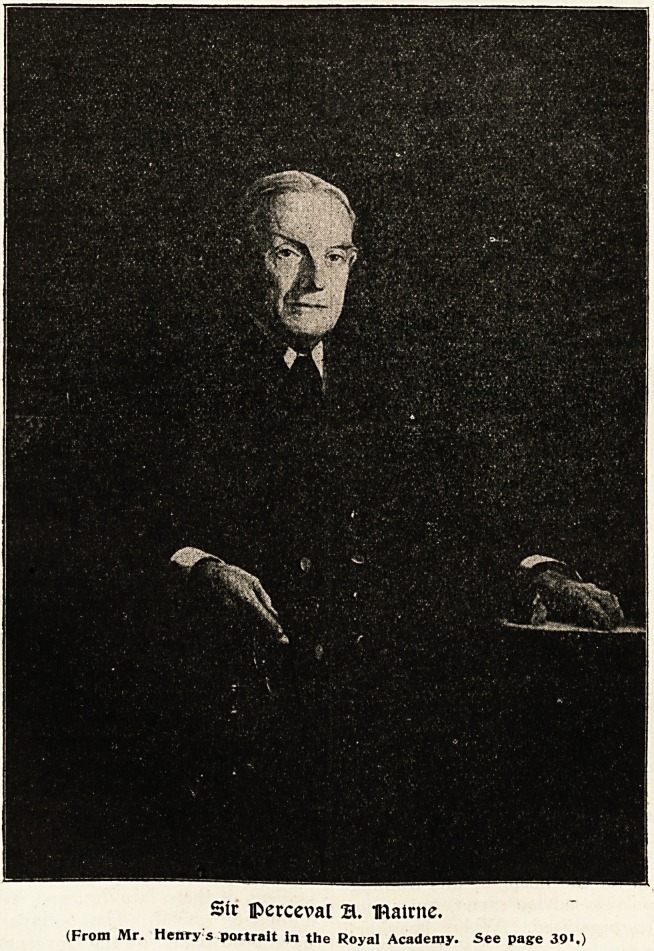# Hospital and Institutional News

**Published:** 1916-07-22

**Authors:** 


### HOSPITAL AND INSTITUTIONAL NEWS.

### DEATH OF SIR VICTOR HORSLEY.

As we go to press we hear news of the death in Mesopotamia of Sir Victor Horsley. This well-known surgeon was, up to quite recently, Colonel-Consultant to the Mediterranean Expeditionary Force, and had, it appears probable, been moved to Mesopotamia in connection with a new scheme of medical arrangements to replace the deficiencies of the previous organisation. Sir Victor, the son of the late J. C. Horsley, R.A., was born at Kensington in 1857, and educated at Cranbrook and the University of London. He qualified M.B. Lond. and B.S. (University Scholar and Gold Medal in Surgery) in 1881, F.R.C.S. Eng. in 1883, was a Fellow of the Royal Society, and received the honour of Knighthood in 1902. His hospital appointments were surgeon to the National Hospital for the Paralysed and Epileptic, Queen Square, and consulting surgeon to University College Hospital. His speciality was surgery of the nervous system, and he was chiefly famous for his operations on the cranium and brain. Sir Victor was a prolific writer on professional subjects, and a member of many learned societies; he was a man of very pronounced opinions on several social questions, including women's suffrage and total alcoholic abstinence, in favour of both of which movements he was a strong protagonist. Sir Victor Horsley leaves a widow, daughter of the late Sir F. J. Bramwell, Bart.

**Sir Perceval A. Nairne.**

(From Mr. Henry's portrait in the Royal Academy. See page 391.)

### THE DEATH OF PROFESSOR METCHNIKOFF.

The death of Professor Elias Metchnikoff, at an advanced age, robs Paris of one who had been in his time a most fertile and ingenious propounder of new ideas in bacteriology and its applications to practical medicine. Of Russian origin, Metchnikoff passed most of his career in Paris, and was thus a precursor, as it were, of the Franco-Russian Alliance. His name will always be remembered in connection with the phagocytic hypothesis, of which he was practically the originator and the most devoted exponent, in contradistinction to the greater importance ascribed by Ehrlich and his school to chemical constituents of the serum. In later days Metchnikoff's brilliancy and originality of conception led him into speculations which, though attractive and fascinating, did not turn out to be as sound as he thought them. The Bulgarian bacillus business, and Metchnikoff's extravagant ideas about the indefinite prolongation of human life, led to a considerable eclipse of his influence; but of his intellect and versatility there was never any question, and undoubtedly his personality and work did much to advance the infant science of bacteriology in the latter years of the last century.

### STAFF VACANCIES AT LONDON HOSPITALS.

The lamented death of Dr. W. W. H. Tate, after a long illness, creates a vacancy in the post of obstetric physician to St. Thomas's Hospital, and lecturer on diseases of women in the medical school there. Dr. Tate was a University College man originally: besides St. Thomas's, several other institutions had at different times the advantage of his services—namely, the Samaritan Hospital, the National Hospital, Queen Square, and the Radium Institute. He examined for the final "Conjoint" for five years. Presumably his death means a step up for each of the junior obstetric visiting staff at St. Thomas's: it is not yet announced whether the vacancy at the foot of the list will be filled now or after the war. It may be noted, however, that several important hospitals are filling their visiting staff vacancies in spite of the war, which may be hard on eligible young men serving abroad. Thus the London Hospital has appointed Dr. Eardley Holland to the vacancy created by the death of Dr. Maxwell; King's College Hospital has appointed Mr. Gilliatt as assistant obstetric surgeon; Guy's Hospital has appointed Mr. Bromley as assistant surgeon; and the Metropolitan has added Mr. Clifford White to its staff—all within the last two or three weeks. We believe it is correct to say that all these surgeons are of military age; we should have preferred to see temporary appointments made from among men of over forty-five.

### A CENTRAL BUREAU FOR ENTERTAINMENTS.

The scheme of entertainments for the wounded, arranged by the Birmingham branch of the British Red Cross Society, with its series of garden parties, as lately described in The Hospital, continues to grow. The organisation has proved itself sufficiently successful to lead to requests from men at the training camps at Aldershot, Salisbury, the Crystal Palace, and Wales for similar fixtures. For instance, on July 29, 2,000 naval men are to be entertained. If the latest appeal for funds be successful it is hoped that a stay may be made at each of the centres above mentioned, so that the men in the various hospitals of each district may also be entertained. The honorary organising secretary of the branch, Mr. Ernest C. Thomas, has arranged for the entertainment of parties varying from 25 to 2,500, and we understand that hints can be obtained on application at the branch office, Queen's College, Paradise Street, Birmingham, on points on which his experience may be of value. If the proposed excursions prove successful, as there is little room to doubt, the Birmingham branch will have developed into a kind of central entertainment bureau for the hospitals and training camps, since not only is advice given, but visits are paid and concerts organised. Where hospitals in the smallest provincial towns feel that their patients are entertained somewhat haphazardly, might not a combined request be made and a local fund raised to lead to a general visit to the neighbourhood?

### DIVERSIFYING A RED CROSS WEEK.

The Flag Day movement, that vigorous child of the original Hospital Sunday idea, is being succeeded by Red Cross weeks in various provincial centres, and it is interesting to see how, for instance, the organisers of the just-ended week at Ipswich arranged their features over the seven days. Running from one Saturday to another, the first day began with a house-to-house distribution of envelopes for contributions. Sunday, apart from pulpit references, was spent in considering how much should be inserted in these envelopes. On Monday they were collected. Then came a Flag Day. Then military sports and tea. On Thursday there was an "American sale" and a public view of the depôt at work, on Friday a morning show of "movies" at the picture-house, a Red Cross auction sale after luncheon, and a fair in the evening, while on Saturday the final rounds of the military sports, a tattoo, and the last day of the fair completed the week. In case these weeks commend themselves generally, we give the above examples of the way in which a week can be filled.

### NOTTINGHAM'S CLAIMS ON RETFORD'S POCKETS

That county towns should be careful in asking smaller places if collections may be held in them on behalf of the county hospital is clear from the criticism which the Nottingham and Notts Hospital Saturday Committee has brought upon itself. This committee asked the Retford Town Council for permission to hold a collection on behalf of the Nottingham General Hospital. In moving that no date be given, to which the meeting agreed, speakers pointed out that Retford never sent patients to Nottingham, which was too far away, that they had to find £700 a year to maintain their own hospital, and that if necessity arose it was to Sheffield or Lincoln that patients were sent. One speaker declared that in ten years he had not received a single application for the Nottingham Hospital, and that the Sheffield Royal Infirmary was the only institution which had any claim upon them outside. This fact, it was explained, and no want of regard for the Nottingham General Hospital, dictated the decision. In face of these facts someone at Nottingham seems to have blundered badly, and exposed its Saturday Committee to ridicule. Who is it?

### THE FAMILY CONDITION OF INTERNED ALIENS.

The Report of the Local Government Board for Scotland contains plenty of evidence that aliens cannot be interned without the creation of problems which affect other public departments. It records, for instance, that considerable distress has existed among the wives and families of interned aliens, with the result that the Parish Councils have been compelled to investigate the circumstances of these families, and, where necessary, to administer relief. The outlays of each Council have been repaid by the Government. In addition, special accommodation has had to be provided for tuberculous and mental cases among the refugees. When the time comes to write the history of public health administration under war conditions, a remarkable chapter will have to deal with the new problems undertaken since the first batch of refugees from Belgium were received and housed at the Alexandra Palace under the control of the

Metropolitan Asylums Board. The above point in the Report of the Local Government Board for Scotland is worth notice at a time when people suppose that once an alien is interned no further public notice need be taken of him and his family.

### INCIDENTS OF AMBULANCE WORK: PELTING THE WOUNDED.

It may seem that the short drive between station and hospital, which our ambulance men have to take when conveying the wounded to the end of their journey, does not offer much scope for adventure. At Liverpool, however, last week, not only on at least one occasion did three heavy trainloads arrive within fifteen hours, but the excitement of the waiting crowds led to various friendly, but untimely, missiles being thrown at the ambulances. One of these was a bloater, about the last thing a wounded man at the end of a long journey would desire to have coming in at him through the window. These missiles, in fact, have proved a real risk to the wounded, and if enthusiasm or gratitude cannot take a less thoughtless form the sufferers can hardly be grateful for it.

### BOARD-ROOM PORTRAITS.

The value of having a gallery of portraits in every hospital board-room has often been insisted on in these columns, because to keep a living tradition of personal service regard must be paid to past examples as well as to the present. It is good news, therefore, to hear that the Norfolk and Norwich Hospital has lately received a portrait of the late Sir Peter Eade, M.D., whose services to the institution were numerous and varied. The portrait, which is the work of Edward Elliott, is not the only permanent memorial of Sir Peter, for his services included historical researches into the past of the institution, certain humorous excerpts from which appeared in The Hospital in a recent issue.

### A LARGE ESTATE FOR SHEFFIELD CHARITIES.

Particulars have appeared in the Press of the will of Miss Mary Eliza Chester, of Sheffield, whose estate is valued at £42,633 gross, with net personalty £38,571. She left the residue of her property to her sister; and, in the event of her sister being dead, she gave such sums as, with what her family have already given, will make up £11,000 to the Jessop Hospital for Women, £11,000 to the Sheffield Royal Hospital, £6,000 to the Free Hospital for Sick Children, £1,000 to the Sheffield Royal Infirmary, and £200 each to five other charities. The ultimate residue goes to the Jessop Hospital, the Royal Infirmary, the Royal Hospital, and the Sick Children's Hospital. The exact wording of these bequests probably makes it clear whether the sums, which these institution have to " bring into account " as received from Miss Chester and her family, include annual subscriptions as well as donations. But if there is any ambiguity on this point the executors and the institutions named may find themselves involved in law proceedings to determine exactly what these provisions of the will are to be taken to mean.

### PRIVATE OPPOSITION TO PUBLIC ECONOMY.

The elimination of waste products is a more popular method of economy than the curtailment of expenditure, but many obstacles are put in the way of individuals who would practise it. Very few towns have adopted the device of sifting their rubbish on the lines that a medical officer of health lately described. Nor have we seen any firm manufacturing the thousand-and-one articles of general consumption sold in tins or glasses offering to purchase these through the shops from those who deal with them. Yet the demand for tin is so great that some firms are giving up its use and substituting wood in place of it; while the waste of tins and glasses is prodigious. Tobacconists might well be glad to obtain these tins again, one would have thought; but they take no steps to do so. Yet, apart from the immense saving that could be effected with the co-operation of the shops in this respect, any diminution of house refuse is a preventive sanitary measure not to be neglected in the interests of public health.

### THE TREATMENT OF VENEREAL DISEASES.

The Local Government Board has issued regulations for putting into effect the recommendations of the Royal Commission on Venereal Diseases. These regulations are made under the Public Health Acts, and it is interesting to note that the war is regarded as a case of emergency within the Act of 1913. The Local Government Board requires local authorities to put the regulations into effect: the scheme is not permissive, that is to say, but obligatory. The Council of every county and county borough must make arrangements by which any doctor practising in the area of the Council can obtain at the Council's expense a pathological report on any case of (suspected) venereal disease. The Council must also provide treatment at or in institutions for sufferers from these diseases, and must supply doctors who have had experience in salvarsan treatment with that drug, or its substitutes, for prevention and cure. The treatment provided must be available for all comers, irrespective of residence or means: the clinics are not to be designated as for venereal diseases, and nothing is to be done to distinguish those who attend them. The Government will contribute three-quarters of the cost of these schemes to the local authorities concerned.

### ECONOMY IN THE ANNUAL REPORT.

In examining many annual reports we have noticed in numerous instances that an effort has been made this year, in view of the increase of cost in printing and of paper, to confine the issue to essentials. We had not, however, until the receipt of the report of the Hampstead General and North-West London Hospital come across a case where what is usually considered the minimum of information necessary in such a publication has not been supplied. The Hampstead Hospital, as it must do to conform with the requirements of the Metropolitan Central Funds, has printed the report of the committee of management, the income and expenditure account, the balance-sheet, the statistical tables, and the list of investments, but has, we understand, obtained a special dispensation to omit this year the list of subscribers and donors. The report in its attenuated form has not been issued, but, as announced by advertisement, is at the disposal of any person who wishes to see it. Somewhat similar procedure has been taken by the Great Northern Central Hospital, where the governors were asked by postcard if they wished a report to be sent to them; a number said they did, but a much larger number congratulated the hospital on the economy effected by a smaller issue, an economy, of course, which refers only to cost of paper and postage, as the report in full (less unessential features) has been printed. We have not yet heard of a provincial hospital restricting its issue of the report, although, we believe, none have Central Fund regulations in this respect, and are influenced only by policy, propriety, and expediency. Unfortunately this is not likely to be the last expensive year; would it be too much to ask for a pronouncement by the King's Fund for guidance as to uniformity in London in respect of next year's report?

### A HOSPITAL FOR THE R.F.C.

AMONG the numerous war hospitals which have come into being all over the country, and especially in London, there are few which are intended to serve the needs of any special section of the Army. The special hospitals for Indian troops were necessitated by religious and racial peculiarities, and have long been closed. Various hospitals for Canadians, South Africans, and other Colonial troops have been established lately, and there is apparently a tendency at the moment towards specialisation of this sort. Quite lately a special hospital has been opened in Lady Tredegar's house in Bryanston Square for the Royal Flying Corps. It is possible, no doubt, to contend that the very special work of this Corps results in certain forms of overstrain and illness which are different from those seen in other branches of the Service; obviously there can be no difference in the nature of the wounds. But on the whole it is doubtful whether this sort of segregation in hospital treatment is advisable or advantageous for the patients.

### A NEW ISOLATION HOSPITAL AT BEXHILL.

THE first part of a scheme for an infectious diseases hospital, adopted some time ago by the Corporation of Bexhill, has recently been completed. This consists of an observation pavilion, a laundry, and sewage disposal works, on a site of five acres at Clinch Green, two miles from the centre of the town. The drainage and sewage disposal works consist of settling tanks, percolating filters, humus tanks, and an irrigation area. The observation pavilion contains wards for two males and two females, together with a duty-room. These portions of the scheme have cost, including furnishing, about £1,150; the estimated cost of the entire hospital is £6,500. The buildings are of brick, with rough-cast and local red-brick dressings, plinths and sills, and have slated roofs, with red-roll ridging. The floors are of polished pitch-pine blocks on concrete. The heating is by open fireplaces, and the lighting by incandescent gas. The grounds have not yet been laid out, but on the completion of the full scheme it is intended to plant shrubs in addition to those trees already there.

### THE LEASOWE OPEN-AIR HOSPITAL.

ALTHOUGH considerably delayed by the war, the Open-Air Hospital for Crippled Children at Leasowe, which is one of the enterprises of the Liverpool Invalid Children's Association, is now nearing completion. The administrative block occupies the centre of the fourteen acres upon which the institution stands, and grouped round are the hospital wards built on the open-air system, with wide sun balconies. Several of these wards were completed before the war broke out, and 150 patients have been in residence since. "Sun treatment" is one of the special features of the hospital, nd already it has proved remarkably satisfactory. The total cost of the institution, inclusive of site, buildings, and equipment, is £48,000, and towards this amount £38,000 has been obtained.

### THE BECKETT DENISON INFIRMARY, DONCASTER.

THE Beckett Denison Infirmary at Doncaster, which is to be erected in the splendid grounds, five or six acres in extent and valued at nearly £10,000, left for that purpose by the lady whose name it bears, will double the hospital accommodation of the town. The present infirmary contains sixty-five beds, but the governors desire that the new one, which will probably cost about £20,000, shall have accommodation for 120 to 150 patients. The old Hall left by Miss Denison will be adapted to the purposes of a nurses' home, and the gardens will be preserved for the patients. The people of Doncaster—and especially the colliery companies and the miners—may be relied upon to provide the increased revenue which the scheme will make necessary. Since the infirmary was enlarged from forty-three to sixty-five beds five years ago the income has never met the expenditure, and the accumulated adverse balance is now £2,700. The present annual income (£4,000) will have to be doubled, or more than doubled, to meet the annual charges when the Beckett Denison Infirmary is an accomplished fact.

### PROGRESS AT THE MIDDLETON SANATORIUM.

SINCE the opening of the sanatorium at Middleton in Wharfedale, in November last, this institution has dealt with 160 cases of phthisis, of which number seventy-six are at present in residence. The members of the West Riding Insurance Committee who visited the sanatorium the other day had a practical demonstration of the advantages that accrue to the early case of consumption when undergoing "sanatorium treatment." It is to be noted that a further extension is contemplated, so that in the near future it is hoped to accommodate

300 patients. It has been found that better results are obtained by increasing the average period of treatment from three to four and a half months, and the experience of the West Riding County Council tallies in this respect with that of other health authorities throughout the country. Nothing is more disheartening either to the patient or to the tuberculosis officer than to find that the former is to be discharged on account of the time-limit just when the treatment is beginning to do good.

### A NEW OPEN-AIR INSTITUTION AT MANCHESTER.

Intended for poor, sickly children who are likely to be cured within a limited period, the Alice Briggs residential open-air school was opened last week. Situated on the top of a hill in fifteen acres of ground at Heaton Mersey, it was a gift to Manchester by Mr. William Briggs and is named after his wife. There is accommodation for fifty children, which later will be doubled. The Edinburgh School Board, which has had under consideration the provision of open-air schools and has appointed a deputation to visit institutions of the kind in England, should include the Alice Briggs school in their list.

### THE TREATMENT OF OPHTHALMIA AT LIVERPOOL.

Most satisfactory is the work which is being accomplished by St. Paul's Eye Hospital, Liverpool, especially in regard to infantile ophthalmia. At the annual meeting of the hospital it was reported that the treatment was so effectual that practically every case was saved if it was only brought at a sufficiently early stage. Out of 161 cases treated last year, 152 were cured absolutely, eight remaining damaged, but not blind. Since this special branch of treatment was undertaken, 1,157 cases have been cured absolutely, and during the last three years, with one solitary exception, there has not been a case of blindness. This is a fine record, upon which the medical staff and all concerned are justly entitled to congratulation. Owing to the continued absence of Lieut.-Colonel A. Nimmo Walker and Lieut.-Colonel Thomas Stevenson, members of the medical staff who are serving at the Front, the hospital has experienced considerable difficulty in carrying out a "business as usual" resolution, and, though the expenditure has been reduced to its lowest limits compatible with efficiency, the growing deficiency, which now amounts to £1,203, is alarming the committee.

### THE ST. PANCRAS SCHOOL FOR MOTHERS.

This well-known, indeed pioneer, infant welfare organisation has lately appealed to the St. Pancras Borough Council for support from the rates. When the school was established, St. Pancras stood twenty-fifth out of the twenty-nine metropolitan boroughs in its infantile mortality. By 1914 it had crept up to ninth; the death-rate among adults showed no improvement corresponding to that among the children, so the St. Pancras School for Mothers is justified in claiming a large share in this most satisfactory improvement. Now that the Councils are empowered to contribute to schemes of this sort, the school finds great difficulty in raising voluntary subscriptions; former subscribers say the school should be supported by the Council. In applying to the Council for a subsidy of £800 to £1,000 a year, a deputation from the school pointed out that Westminster pays £500 and Leeds £1,600 annually for their respective schools. The application has been referred to the Parliamentary Committee of the Council, with what result remains to be seen.

### CONSUMPTION IN COTTAGE HOMES.

In recording the fact that thirty-five fresh cases of tuberculosis had been notified in Pembroke, Dr. Morgan emphasises the fact that several of them had occurred in families previously attacked by the disease. He declares that since the County Council have failed to proceed with their tuberculosis scheme, only the well-to-do and the insured can obtain sanatorium treatment. A tuberculosis hospital was especially needed in this district owing to the large number of three- and four-roomed cottages, in which, Dr. Morgan remarks, it is almost impossible to prevent the infection of other inmates. What is still worse is the absence of institutional treatment for advanced cases, which thus unprovided for return to become centres of infection. We believe that this cardinal defect is far more responsible for the spread of disease than the cottages which Dr. Morgan naturally regards as a source of danger.

### ALLOTMENTS AND THE PUBLIC HEALTH.

We are accustomed to attribute improvements in the health of villages and towns to the public health services, and no one contests this view. But there are other factors less regarded, but also helpful. For instance, in an elaborate parochial history, compiled by a medical man who spent his leisure in antiquarian research, we find the following remarkable tribute paid to the value from a public health point of view of allotment gardens. In the village of which he wrote these allotments were first provided about forty years ago, and this is the doctor's verdict upon them: "The introduction of allotments away from the village has been of considerable advantage, in a sanitary point, to the health of the villagers." The annual visitation of fever was due, he says, "to the small gardens at the backs of the houses being crowded in the arrangement of the pig-sties, closets, and muck-heaps, which trenched too closely on the back doors." Then in 1875 sanitary officers were created, and refuse materials "found their way chiefly to the allotment gardens, and thus contamination of the water in the shallow wells or dip-holes was prevented, to the comparative exclusion of a long-standing menace to the health of the population." Thus the increase in the amount of land available for the cottagers, their enrichment in fact, has proved, as is always the case in the long run, a sanitary reform.

### THE SALE OF MEDICATED WINES.

An Order in Council has been gazetted which places a long-needed restriction on the sale of medicated wines. The Order, which comes into force on August 7, provides that no person may supply in any licensed premises for consumption off the premises any intoxicating liquor in the form of medicated wine, or any mixture or preparation containing any drug or medicament sold or advertised as containing medicinal properties except in a vessel bearing a label stating clearly the amount of proof spirit it contains. The Order does not apply to any preparation appearing in the British Pharmacopœia or the British Pharmaceutical Codex, signed prescriptions of a medical practitioner, or medicated or methylated spirits or spirits made up in medicine and sold by medical practitioners or chemists and druggists. It might appear to the casual observer that the exceptions to the Order almost nullify the effect of the Order, but this is not the case. In order to be able to sell the ordinary medicated wines of commerce the chemist is obliged to have a licence, and the shops of such chemists are therefore, we take it, "licensed premises." Thus, although the embargo does not prevent a pharmacist from continuing to sell tinctures and other ordinary spirituous medicines, as in the past, it will make it necessary for him to state on the label the amount of alcohol contained in the advertised medicated wines in which such an extensive—in our view far too extensive—business is done. Just as medicines containing morphine and cocaine are in some measure responsible for the spread of the drug habit in its most pernicious forms, so the habit of drinking stronger liquors is sometimes acquired as a result of drinking medicated wines, which often contain a minimum of medicine and a maximum of tenth-rate wine. Whether the knowledge that a particular brand of "medicated wine" contains a certain definite percentage of alcohol will deter people from buying the wine remains to be seen, but it is clearly in the public interest that people should know what they are drinking.

### THE ROYAL MIDLAND INSTITUTION FOR THE BLIND, NOTTINGHAM.

Presiding at the annual meeting of the Royal Midland Institution for the Blind, Nottingham, Earl Manvers spoke of the report presented as the most satisfactory he had ever seen in his long association with the institution. The number of blind persons now connected with the institution is 125, of whom forty-five are workers and eighty pupils; seventeen who have passed out are living lives of useful industry. The sales reached the highest point ever attained, viz. £10,402, and the total amount received by blind persons in the way of wages, £1,770, was likewise a record. However, there was the inevitable fly in the ointment—a diminution in the subscriptions. Lord Manvers did not believe the subscriptions would continue to decrease, for help for the blind was needed in these days more than ever before, and an institution which benefited the country so enormously could not permanently suffer. Canon Holbrook said he feared the public sometimes looked upon the institution as receiving constant gifts and grants from a bounteous Government.

### A MAN OF MANY PARTS.

Such male employees as the war has left to the institutions have to turn their hands often to unfamiliar tasks and literally make themselves "generally useful." It is not frequently, however, that a male servant is required to undertake such varied duties as the labour master for whom the Guardians of the Faversham Union are advertising in the *Local Government Chronicle*. Candidates for the vacant post, who must be ineligible for military service, are required to do the hair-cutting and shaving of the inmates, superintend their employment and the cultivation of eleven acres of land; they must also have a thorough knowledge of gardening, and understand the working of a small petrol engine for sawing wood. The salary of this protean officer is not stated.

### THIS WEEK'S DRUG MARKET.

Important fluctuations in prices have not been numerous. English makers of quinine announce an advance in their quotations; the advance, however, is not substantial, and makers' normal prices are still below those quoted for Continental brands. All the salts of mercury, with the exception of corrosive sublimate, are dearer in consequence of the advance in the price of the metal. Salicylic acid is again lower in price, and it is quite probable that the lowest figure has not yet been reached; salicylate of soda has not receded in price to quite the same extent. Acetylsalicylic acid, acetanilide, and hexamine also have a slightly downward tendency in value. On the other hand, phenacetin is again dearer. The market for camphor is now quieter, but the higher price to which Japanese refined had advanced is maintained. There is no change in the position of potassium bromide and other salts of bromine. The price of tartaric acid is not so firmly maintained, but there are no signs of the value being likely to recede to any substantial extent. Cod-liver oil continues to tend downwards in value, but the price is still a very long way from anything like a reasonable figure.

### TO OUR READERS.

**Contributions are specially invited from any of our readers to these columns. They should deal with topical subjects and news. They must be authenticated for the information of the Editor only. The minimum payment if published is 5s. There is no hard-and-fast rule as to space, but notes of about twenty lines in length are preferred.**

## Figures and Tables

**Figure f1:**